# Friendship of Doctors

**Published:** 1884-01-20

**Authors:** 


### *FRIENDSHIP OF DOCTORS.*

There is no class of people who have greater reason for cultivating kindly relations, the one for the other, than medical men.

It is not only dishonorable and wrong to speak disparagingly of a medical brother, but as a matter of policy it is bad, and works, in the end, injury to the party who does it, and hurts the profession at large.

In every community the Doctors should organize a society and meet socially together at least once a month, or oftener, to cultivate friendly and social feelings, and to talk over their cases and professional experiences. Try it, friends, it will do you good. You will find that the meetings will be instructive, pleasant and agreeable, and that you will feel and act more liberally toward the brethren, and that your mutual interest and happiness as physicians, will he greatly promoted.

